# Inhibiting HMGCR represses stemness and metastasis of hepatocellular carcinoma via Hedgehog signaling

**DOI:** 10.1016/j.gendis.2024.101285

**Published:** 2024-04-03

**Authors:** Zhirong Zhang, Jiayao Yang, Rui Liu, Jing Ma, Kai Wang, Xiaojun Wang, Ni Tang

**Affiliations:** a Key Laboratory of Molecular Biology for Infectious Diseases (Ministry of Education), Institute for Viral Hepatitis, Department of Infectious Diseases, The Second Affiliated Hospital, Chongqing Medical University, Chongqing 400016, China; b Institute of Hepatobiliary Surgery, Southwest Hospital, Third Military Medical University (Army Medical University), Chongqing 400038, China

**Keywords:** Cancer stem cells, Hedgehog, Hepatocellular carcinoma, Metastasis, Statin

## Abstract

Cancer stem cells (CSCs) play a crucial role in tumor initiation, recurrence, metastasis, and drug resistance. However, the current understanding of CSCs in hepatocellular carcinoma (HCC) remains incomplete. Through a comprehensive analysis of the database, it has been observed that 3-hydroxy-3-methylglutaryl-coenzyme A reductase (HMGCR), a critical enzyme involved in cholesterol synthesis, is up-regulated in HCC tissues and liver CSCs. Moreover, high expression of HMGCR is associated with a poor prognosis in patients with HCC. Functionally, HMGCR promotes the stemness and metastasis of HCC both *in vitro* and *in vivo*. By screening various signaling pathway inhibitors, we have determined that HMGCR regulates stemness and metastasis by activating the Hedgehog signaling in HCC. Mechanistically, HMGCR positively correlates with the expression of the Smoothened receptor and facilitates the nuclear translocation of the transcriptional activator GLI family zinc finger 1. Inhibition of the Hedgehog pathway can reverse the stimulatory effects of HMGCR on stemness and metastasis in HCC. Notably, simvastatin, an FDA-approved cholesterol-lowering drug, has been shown to inhibit stemness and metastasis of HCC by targeting HMGCR. Taken together, our findings suggest that HMGCR promotes the regeneration and metastasis of HCC through the activation of Hedgehog signaling, and simvastatin holds the potential for clinical suppression of HCC metastasis.

## Introduction

Hepatocellular carcinoma (HCC) is a globally prevalent malignancy. According to epidemiological investigation, the incidence of HCC ranks fifth, while the mortality rate was third in cancers worldwide.^1^ This inconsistency between incidence and mortality is rooted in the high recurrence, high metastasis, and gradual insensitivity to targeted drugs of HCC.^2^ Notably, distant metastasis of HCC, especially pulmonary metastasis accounting for about 47% of cases, is a major factor in patient fatalities.^3^ A wide range of therapeutic options exist for HCC patients, including liver transplantation, surgical resection, percutaneous ablation, radiotherapy, and molecularly targeted drugs such as tyrosine inhibitors.^4^ However, due to the obstacle of early diagnosis, HCC is often detected at an advanced stage with metastasis, resulting in missed opportunities for effective treatment and a poor prognosis. In addition, the recurrence of HCC after therapy is a typical clinical issue, the recurrence of HCC after loco-regional treatments such as resection is approximately 70% of the rate, and the transplantation is about 13%.^5^^,^^6^ The above dilemma makes the diagnosis and treatment of HCC a major global healthcare challenge, thus, original predictive biomarkers and treatment strategies for metastatic HCC are urgently needed.

Cancer stem cells (CSCs) are a distinct subpopulation with abilities of self-renewal and differentiation potential that exist in numerous cancers. A small percentage of CSCs are sufficient for tumor recurrence, metastasis, and drug resistance. Therefore, targeting CSCs is an attractive approach to prevent tumor recurrence and metastasis, in contrast to conventional therapies targeting the bulk tumor cells.^7^ There also exists a group of CSCs in HCC with surface markers such as CD13, CD133, EpCAM, CD44, CD24, CD90, and NANOG identified as contributors to the maintenance of self-renewal.^8^ Epithelial-mesenchymal transition acts as the initial phase of metastasis, facilitating the acquisition of mesenchymal attributes by epithelial cells. Generally, this process contributes to the production of CSCs and its related markers often elevate expression levels in CSCs. In turn, CSCs acquire the ability to metastasize more easily than bulk tumor cells. This may explain the prolonged invasiveness of CSCs after metastasizing to distant places.^9^

Cholesterol synthesis plays a crucial role in cancer metabolism, with 3-hydroxy-3-methylglutaryl-coenzyme A reductase (HMGCR) serving as the first key rate-limiting enzyme in this process.^10^ In general, HMGCR promotes cancer progression mainly through the geranylgeranyl pyrophosphate (GGPP), a downstream metabolite of HMGCR, which regulates the small GTPase family proteins by prenylation modification to induce apoptosis and cell cycle arrest in cancer cells.^11^^,^^12^ Previous studies have indicated that a variety of metabolic enzymes in cholesterol synthesis are involved in the persistence of tumor stemness, including breast cancer, bladder cancer, and colon cancer.^13^, ^14^, ^15^ However, the impact of HMGCR on the stemness and metastasis of HCC as well as the underlying mechanisms have not been fully uncovered. In addition, statins, a targeted inhibitor of HMGCR, is an FDA-approved clinical cholesterol-lowering drug. Its drug pleiotropy has been extensively dug up. Statins can act as cancer suppressors in several ways, mainly by inducing apoptosis and cell cycle arrest.^16^^,^^17^ Nevertheless, whether statins can weaken tumor progression and metastasis by reducing tumor stemness in HCC is still a puzzle.

Herein, we hope to reveal the role of HMGCR in the metastasis of liver CSCs and the underlying mechanism specifically. The results showed that HMGCR contributed to the maintenance of HCC stemness and promoted metastasis through activating the Hedgehog signaling. Moreover, simvastatin provided a novel clinical choice to suppress the metastasis of HCC by targeting the liver CSCs.

## Materials and methods

### Patient samples

The paired HCC tissues and adjacent non-tumor tissues were acquired from a cohort of 42 HCC patients who underwent liver surgery at the Second Affiliated Hospital of Chongqing Medical University. Prior to surgery, written informed consent was obtained from all included patients. This part was approved by the Institutional Ethical Review Board of Chongqing Medical University (No. 22023079).

### Public database analysis

Transcriptomic data and clinical data of 374 patients with HCC were obtained from The Cancer Genome Atlas Liver Hepatocellular Carcinoma (TCGA-LIHC) through the official website of the National Cancer Institute (https://www.cancer.gov/ccg/research/genome-sequencing/tcga). The GSE23034, GSE39791, and GSE5975 datasets were obtained from the GEO database (https://www.ncbi.nlm.nih.gov/geo/).

### Cell culture and treatment

Cell lines SNU449 and PLC/PRF/5 were acquired from the American Type Culture Collection (ATCC, VA, USA), and MHCC-97H, Huh7, HEK293, and HEK293T cells were obtained from the Cell Bank of the Chinese Academy of Sciences (Shanghai, China). MIHA was gifted by Dr. Ben C.B. Ko (Hong Kong Polytechnic University, Shanghai, China). SNU449 was cultured in RMPI-1640 medium (Gibco, NY, USA) supplemented with 10% fetal bovine serum (Natocor, Argentina) and 1% penicillin-streptomycin (MCE, NJ, USA). MIHA, Huh7, PLC/PRF/5, MHCC97-H, and HEK293T cells were cultured in DMEM medium (Gibco) supplemented with 10% fetal bovine serum (Natocor) and 1% penicillin-streptomycin (MCE). To assess the effects of simvastatin, cells were treated with either 15 μM or 25 μM simvastatin (HY-17502, MCE) dissolved in dimethyl sulfoxide. For pathway inhibitor treatment, the concentration and administration time is as follow: verteporfin (HY–B0146, MCE), 5 μM, 36 h; SB431542 (S1067, Selleck, TX, USA), 10 μM, 36 h; XAV-939 (HY-15147, MCE), 10 μM, 36 h; RO4929097 (HY-11102, MCE), 10 μM, 36 h; vismodegib (HY-10440, MCE), 10 μM, 36 h.

### Adenovirus production

The full-length cDNA of HMGCR (coding sequence of NM_ 000859.3) amplified from the total cDNA of hepatoma cells was ligated into pAdTrack-TO4 vectors (Table S1). As described previously, the generation of recombinant viruses of AdHMGCR was performed using the AdEasy system.^18^ The pAdTrack-TO4 plasmid and adenovirus negative control AdGFP were kindly provided by Dr. TongChuan He (University of Chicago, IL, USA).

### Lentivirus infection

To achieve stable interference of HMGCR expression, short hairpin RNA targeting HMGCR (Table S1) was designed and inserted into the lentiviral vector pLL3.7 (Prof. Bing Sun, the Shanghai Institute of Biochemistry and Cell Biology, Chinese Academy of Sciences, Shanghai, China). The CRISPR/Cas9 system was used for SMO (Smoothened) knockout. Single guide RNA sequences targeting SMO were designed (Table S1) and cloned into the lentiviral vector CRISPRv2 (Dr. Ding Xue, Tsinghua University, Beijing, China). Then, shHMGCR lentivirus and sgSMO lentivirus were generated and packaged in HEK293T cells using Lipo8000 (Beyotime, Shanghai, China) as previously described.^18^

### Western blotting analysis

The protein samples were extracted and their concentrations were determined using the protocols described in a previous study.^19^ Subsequently, the protein samples were separated by SDS-PAGE and transferred onto the PVDF membranes. After being blocked by 3%–5% skim milk, primary antibodies were incubated at 4 °C overnight. Then corresponding goat anti-mouse or goat anti-rabbit antibody (Biorad, CA, USA) was incubated in the PVDF membrane for 2 h. Protein signals were visualized using an enhanced chemiluminescence substrate. Primary antibodies and the proportions are as follows: HMGCR (1:2000, PTM-6018, PTM-BIO, Hangzhou, China), E-cadherin (1:1000, ab40772, Abcam, Cambs, UK), N-cadherin (1:1000, BS72312, Bioworld Technology, MN, USA), SMO (1:2000, 66851-1-Ig, Proteintech, IL, USA), GLI1 (GLI family zinc finger 1; 1:500, 66905-1-Ig, Proteintech), β-actin (1:4000, TA-09, ZSGB-BIO, Beijing, China), H3 (1:2000, 100005-MM01, Sino Biological, Beijing, China), and β-Tublin (1:2000, 66240-1-Ig, Proteintech).

### Quantitative reverse transcription PCR

Total cellular RNA was extracted by RNAiso Plus Reagent (Takara, Kyoto, Japan). For cDNA reverse-transcription and genomic DNA elimination, the PrimeScript™ RT Reagent Kit with gDNA Eraser (Takara) was used. The quantitative real-time PCR assay was carried out using an SYBR Green qRT-PCR Master Mix (US EVERBRIGHT, Suzhou, China). Target sequences of the primer are provided in Table S1. All quantitative reverse transcription PCR experiments were performed on the QuantStudio 6 Flex system (Thermo Fisher Scientific, MA, USA). The transcript levels of target genes were normalized to β-actin using the ΔΔCt method.

### Immunohistochemistry assay

All tissues used for staining were pretreated with paraffin embedding. The detection system (ZSGB-Bio) and DAB color development system (ZSGB-Bio) were used for immunohistochemistry assays following the previously described protocol.^20^ The primary antibody HMGCR (1:100, 66905-1-Ig, Proteintech) was used in this study. The images were scanned by Pannoramic Viewer 1.15.2 (3DHistech).

### Immunofluorescence assay

The cell slides were fixed with 4% paraformaldehyde for 30 min and then permeabilized with TritonX-100 (Sigma, MA, USA) for 15 min. Then, the slides were blocked with goat serum (ZSGB-Bio) for 1h. After incubation with the primary antibody GLI1 (1:50, 66905-1-Ig, Proteintech) and incubation with fluorescence-labeled secondary antibody goat anti-mouse IgG/TRITC (ZSGB-Bio), the nuclear was dyed with DAPI (Roche Diagnostics GmbH, Swiss, Germany) at concentrations recommended by the manufacturer. Images were visualized by a laser-scanning confocal microscope (Leica TCS SP8, Wetzlar, Germany).

### Spheroid formation assay

A total of 5000 cells were seeded into a 6-well ultralow attachment plate (Corning, NY, USA) and cultured in DMEM/F12 1:1 medium (Hyclone, UT, USA) with 10% fetal bovine serum (Natocor), 20 ng/mL human recombinant EGF (PeproTech, Nanjing, China), 20 ng/mL human FGF-basic recombinant protein (PeproTech), and 2% B27 supplement (Gibco). After 10 days of culture, the number of low-differentiation spheroids with a diameter greater than 70 μm were counted. As for simvastatin treatment in spheroid formation, 5 μM or 10 μM simvastatin was added into the system, and the inhibitor was replenished after five days of culture.

### Flow cytometry

After digested and isolated into single cells, the cells were stained with PE anti-human CD326 (EpCAM) antibody and APC anti-human CD133 antibody (Biolegend, CA, USA) at 4 °C for 30 min. Flow cytometry was conducted using CytoFLEX (Beckman Coulter, CA, USA). The software Flowjo (ver. 10.7, Tree Star Inc.) was used for data analysis.

### Transwell migration assay

Cell migration was performed using a transwell insert with 8.0 μm pores (Corning). MHCC-97H (4 × 10^4^), Huh7 (4 × 10^4^), PLC/PRF/5 (3 × 10^4^), or SNU-449 (3 × 10^4^) cells were seeded onto the upper compartment in serum-free medium. The lower compartment was replete with a medium containing 20% fetal bovine serum (Natocor). The migrated cells were subsequently fixed with 4% paraformaldehyde and stained with crystal violet (Beyotime). For each experiment, the mean of migrated cells in five random fields was calculated.

### Wound healing assay

Cells were cultured in 96-well plates until confluent, and wounds were created using WoundMaker™ (Essen Bioscience, MI, USA) on the cell monolayer. The real-time wound areas were recorded by the IncuCyte ZOOM Live-Cell Imaging system (Essen BioScience).

### Animal models and treatment

BALB/c nude mice (male, 5 weeks old) were acquired from Cavens Experimental Animal Company (Changzhou, China). MHCC-97H cells were treated with either shHMGCR lentivirus or control lentivirus for 48 h. Then, cells were digested and resuspended in phosphate-buffered saline into a single-cell suspension. The cells were diluted to a series of limited concentrations of 1 × 10^3^, 1 × 10^4^, and 1 × 10^5^ in 100 μL phosphate-buffered saline and subcutaneously injected into the axilla of nude mice. Mice were sacrificed 4 weeks after cell injection, and their tumors were isolated. Each group contained 7 mice.

HCC metastatic models via tail-vein injection adopted six-week-old male BALB/c nude mice. Initially, SNU449 cells were infected with AdGFP or AdHMGCR adenovirus for 48 h. Then, 2 × 10^6^ SNU449 cells suspended in 100 μL phosphate-buffered saline were injected into the lateral tail veins of nude mice. Specific groups can be referred to Figure 5, Figure 7, and mice were included in each group. The mice were sacrificed 10 weeks later, and their lungs were collected for histological examination. For the inhibitor treatment *in vivo*, the mice were intraperitoneally administered simvastatin (10 mg/kg/day) or vismodegib (20 mg/kg/day) for 7 weeks. All animal models mentioned above were approved by the Research Ethics Committee of Chongqing Medical University (No. IACUC-CQMU-2023-0192).

**Figure 1:**
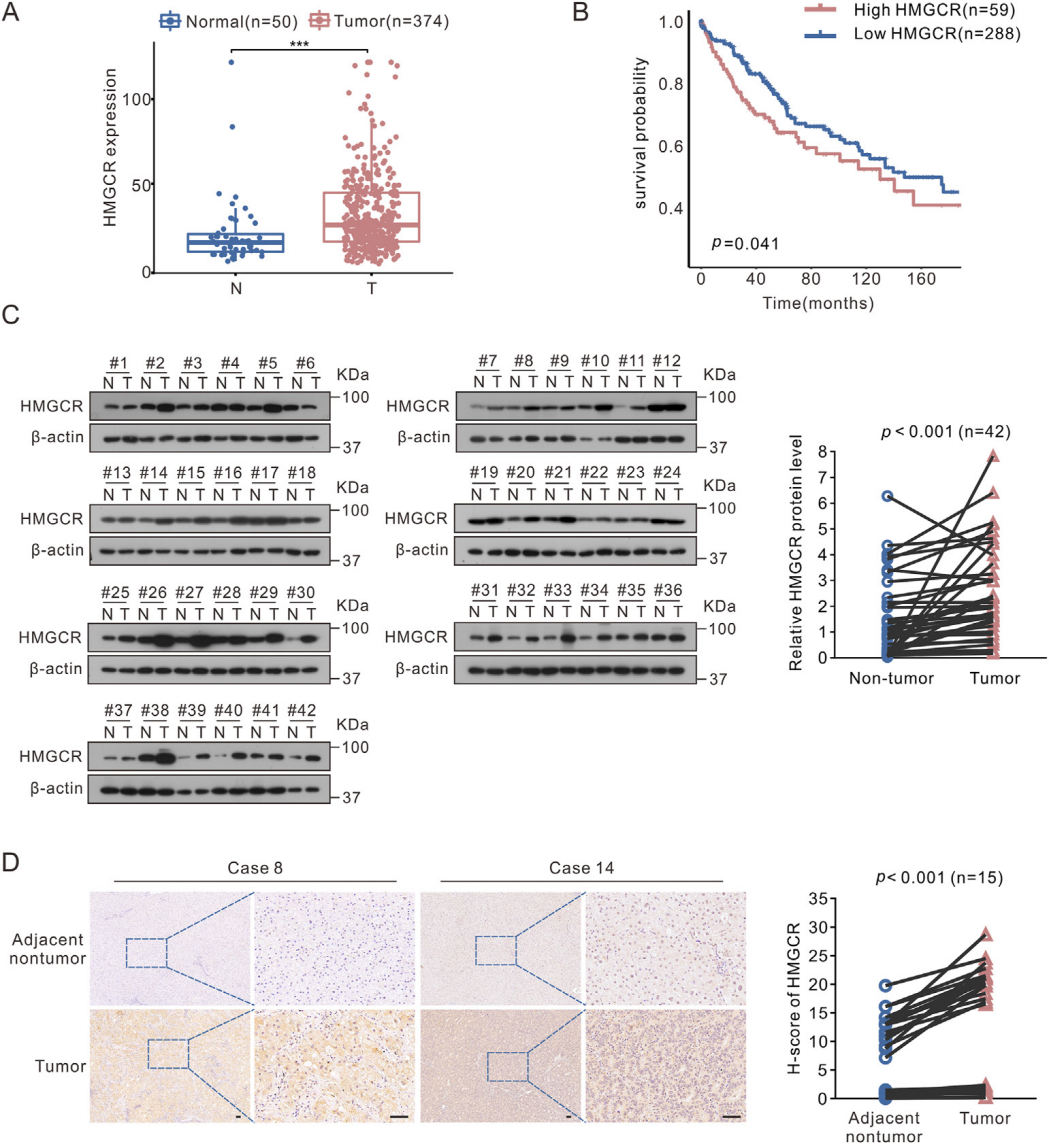
HMGCR was highly expressed in hepatocellular carcinoma (HCC) and associated with poor prognosis. **(A)** Expression of HMGCR at the transcription level between HCC and normal liver tissues in TCGA LIHC dataset. **(B)** Kaplan–Meier overall survival curve based on HMGCR mRNA expression in TCGA LIHC dataset. **(C)** Immunoblot assay for HMGCR expression of 42 paired HCC tissues (T) and adjacent nontumor tissues (N). Quantitative statistical analysis of HMGCR protein expression was normalized to β-actin levels using ImageJ software. **(D)** Representative images of immunohistochemistry staining of HMGCR in clinical HCC samples (*n* = 15). Scale bar: 50 μm. The complete images can be seen in Figure S1A. Statistical analysis of immunohistochemical score on the right. Data are shown as mean ± standard deviation. ^∗∗∗^*P* < 0.001. Differences were tested using two sample *t*-test for (A), log-rank test for (B), and two-tailed paired *t*-test for (C, D).

**Figure 2:**
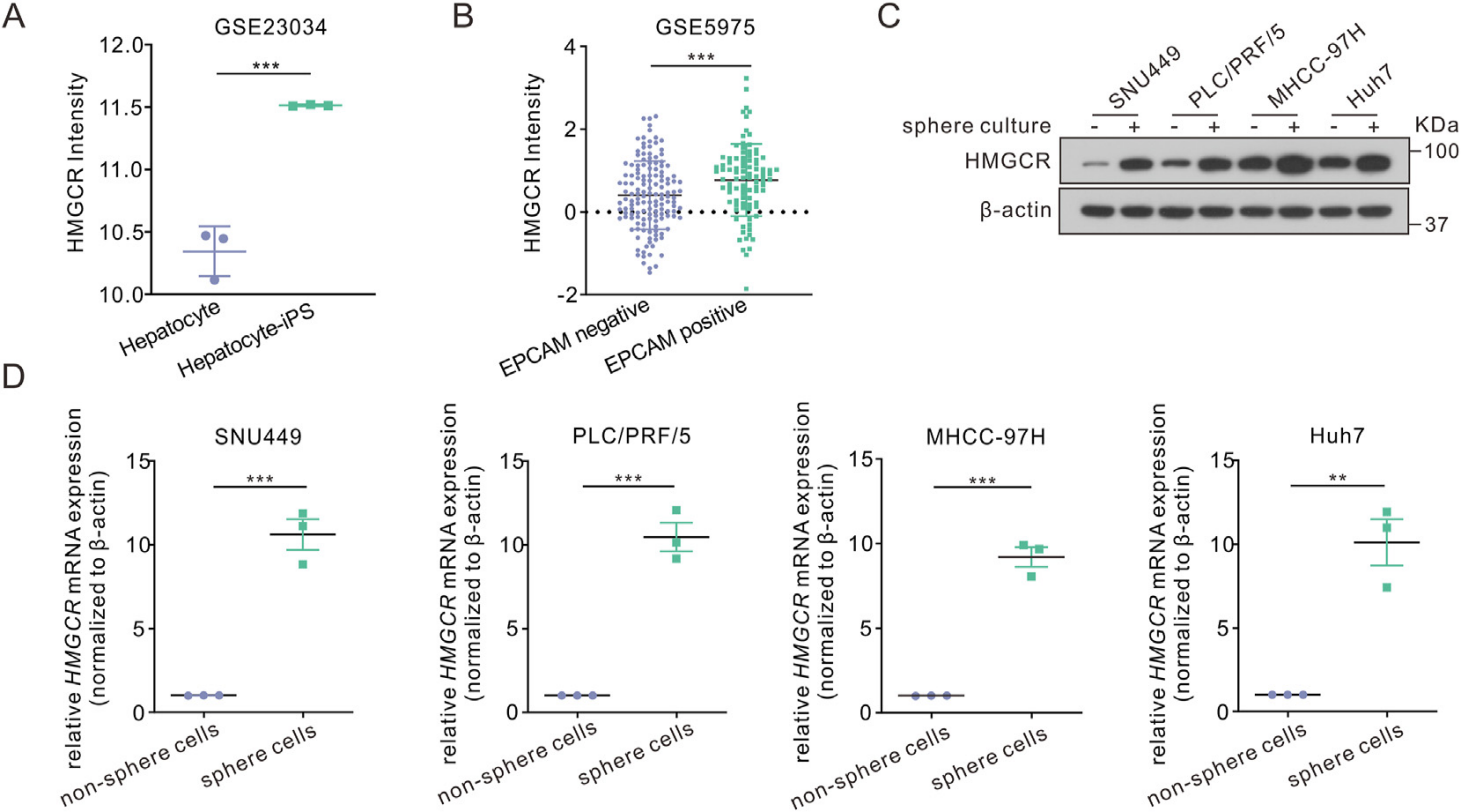
HMGCR was up-regulated in enriched liver cancer stem cell populations. **(A, B)** HMGCR mRNA levels in induced pluripotent hepatocytes and normal hepatocytes in GSE23034 (A), or in EpCAM positive and negative populations in GSE5975 (B). **(C, D)** HMGCR mRNA (C) or protein (D) expression under non-sphere culture or sphere culture in hepatoma cells (SNU449, PLC/PRF/5, MHCC-97H, Huh7). Data are shown as mean ± standard deviation. ^∗∗^*P* < 0.01, ^∗∗∗^*P* < 0.001. Differences were tested using two-sample *t*-test for (A, B, D).

**Figure 3:**
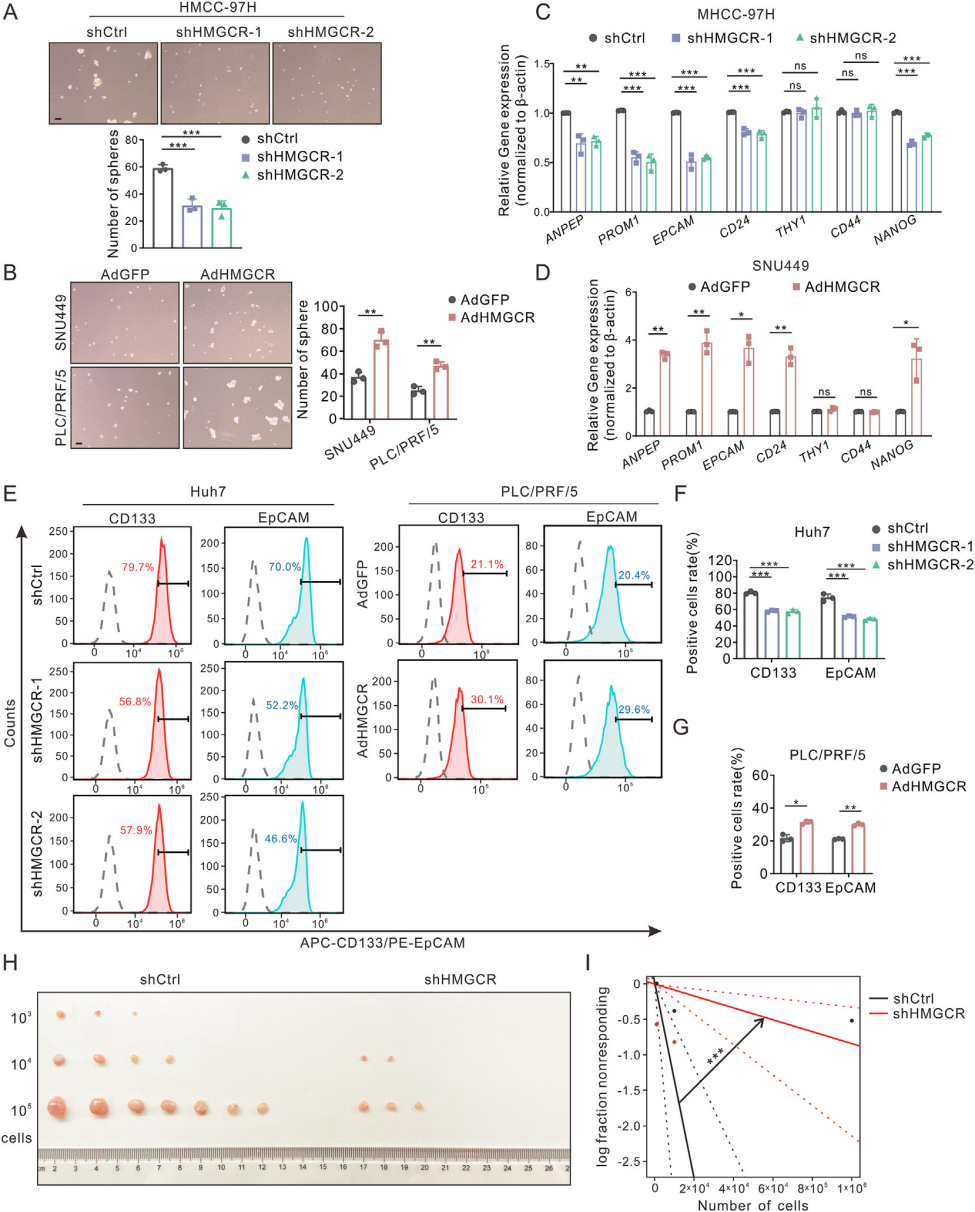
HMGCR promoted stemness features of hepatoma cells. **(A, B)** Representative images of tumor spheres treated as indicated after 10 days of sphere formation culture. The number of hepato-spheres larger than 70 μm in diameter was calculated. Scale bar: 100 μm. **(C, D)** Liver cancer stem cell markers (*ANPEP*, *PROM1*, *EPCAM*, *CD24*, *CD44*, *THY1*, *NANOG*) were determined by quantitative reverse transcription PCR in HMGCR knock-down or overexpression cells. **(E**–**G)** The populations of CD133^+^ and EpCAM^+^ in HMGCR knock-down or overexpression hepatoma cells by flow cytometry. The statistical analysis was shown as percentages in (F, G). **(H, I)***In vivo* limiting dilution xenograft formation of HMGCR knock-down or control MHCC-97H cells (*n* = 7). The tumor gross images (H) and statistical analysis (I) of xenograft tumorigenicity were presented. Data are shown as mean ± standard deviation. ns, not significant; ^∗^*P* < 0.05, ^∗∗^*P* < 0.01, ^∗∗∗^*P* < 0.001. Differences were tested using one-way ANOVA for (A, C, F) and two sample *t*-test for (B, D, G).

**Figure 4:**
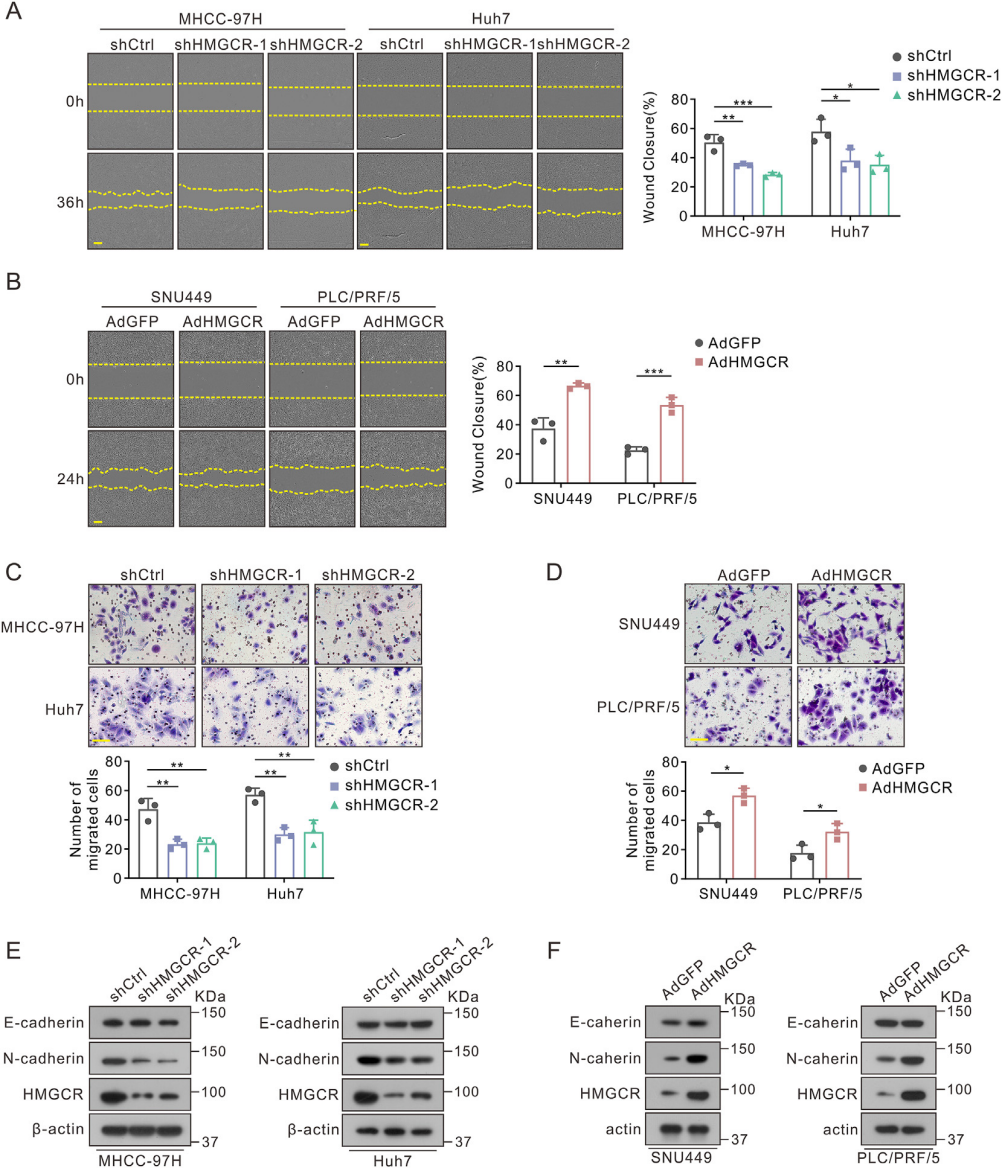
HMGCR facilitated the metastasis of hepatoma cells. **(A, B)** Wound healing assays of HMGCR-knockdown MHCC-97H and Huh7 cells (A) or HMGCR-overexpressing SNU449 and PLC/PRF/5 cells (B). The wound closure percentage is shown on the right. Scale bar: 200 μm. **(C, D)** Representative images of transwell migration assays and quantification of the migrated cells in HMGCR knock-down (C) or overexpression (D) hepatoma cells. Scale bar: 100 μm. **(E, F)** Protein levels of epithelial-mesenchymal-transition-related markers in HMGCR knock-down (E) or overexpression hepatoma cells (F). Data are shown as mean ± standard deviation. ^∗^*P* < 0.05, ^∗∗^*P* < 0.01, ^∗∗∗^*P* < 0.001. Differences were tested using one-way ANOVA for (A, C) and two sample *t*-test for (B, D).

**Figure 5:**
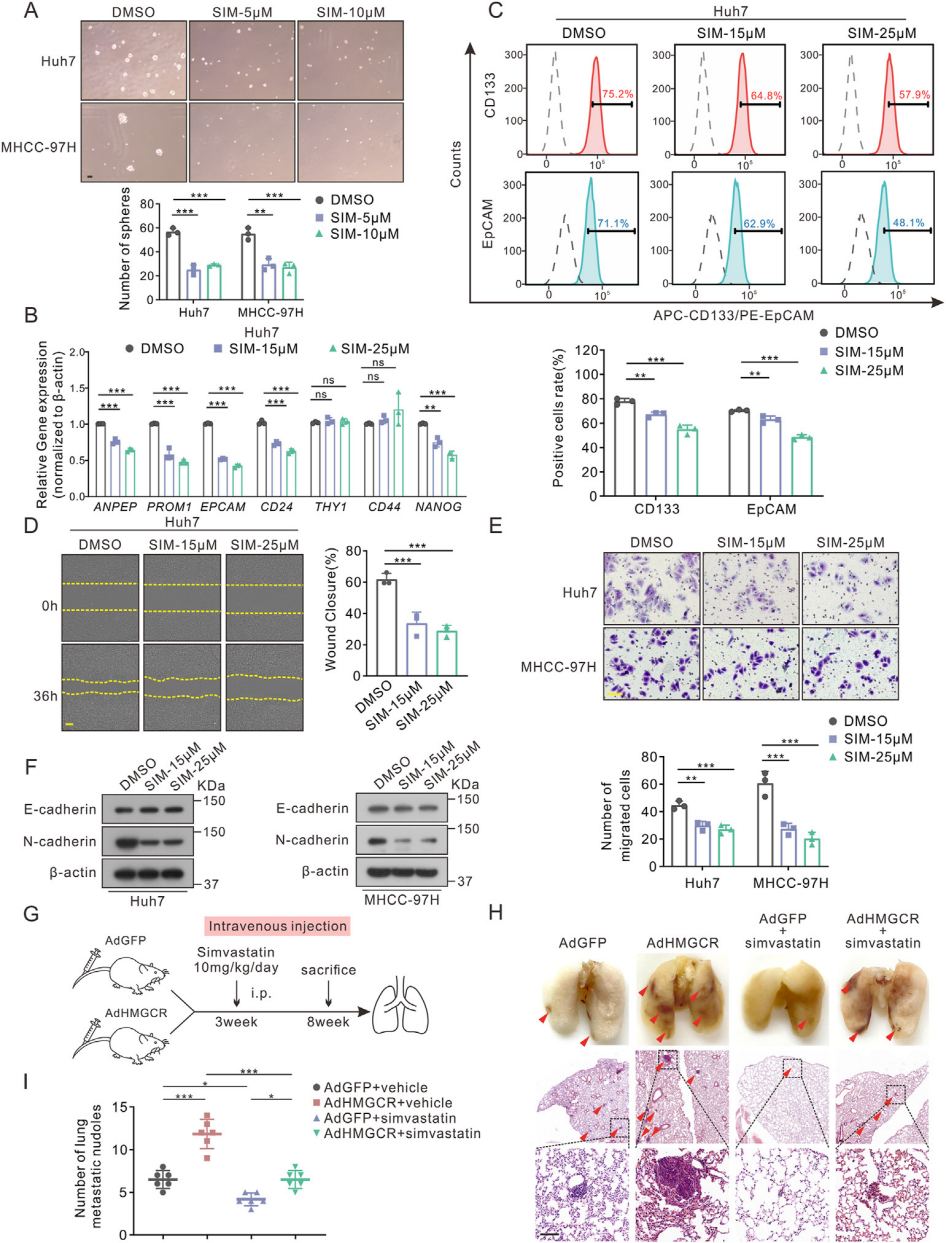
Pharmacological inhibition of HMGCR impaired stemness and metastasis of hepatoma cells. **(A)** Sphere formation assays were conducted for 10 days. Scale bar: 100 μm. **(B)** Stemness-related markers were quantified by quantitative reverse transcription PCR with simvastatin treatment for 36 h. **(C)** The populations of CD133^+^ and EpCAM^+^ Huh7 cells treated with simvastatin were quantified by flow cytometry. **(D, E)** Representative images and quantified results of the wound-healing (D) and transwell assays (E) with or without simvastatin treatment. Scale bar: 100 μm in transwell and 200 μm in wound-healing assays. **(F)** The expression of epithelial-mesenchymal-transition-related markers in Huh7 and MHCC-97H with simvastatin treatment for 36 h. **(G**–**I)** The groups, treatment of tail intravenous injection model (G), and hematoxylin-eosin staining of lung tissues (H). Scale bar: 100 μm. The number of lung metastatic nodules is shown in (I) (*n* = 6). Data are shown as mean ± standard deviation. ns, not significant; ^∗^*P* < 0.05, ^∗∗^*P* < 0.01, ^∗∗∗^*P* < 0.001. Differences were tested using one-way ANOVA for (A–E, I). SIM: simvastatin.

**Figure 6:**
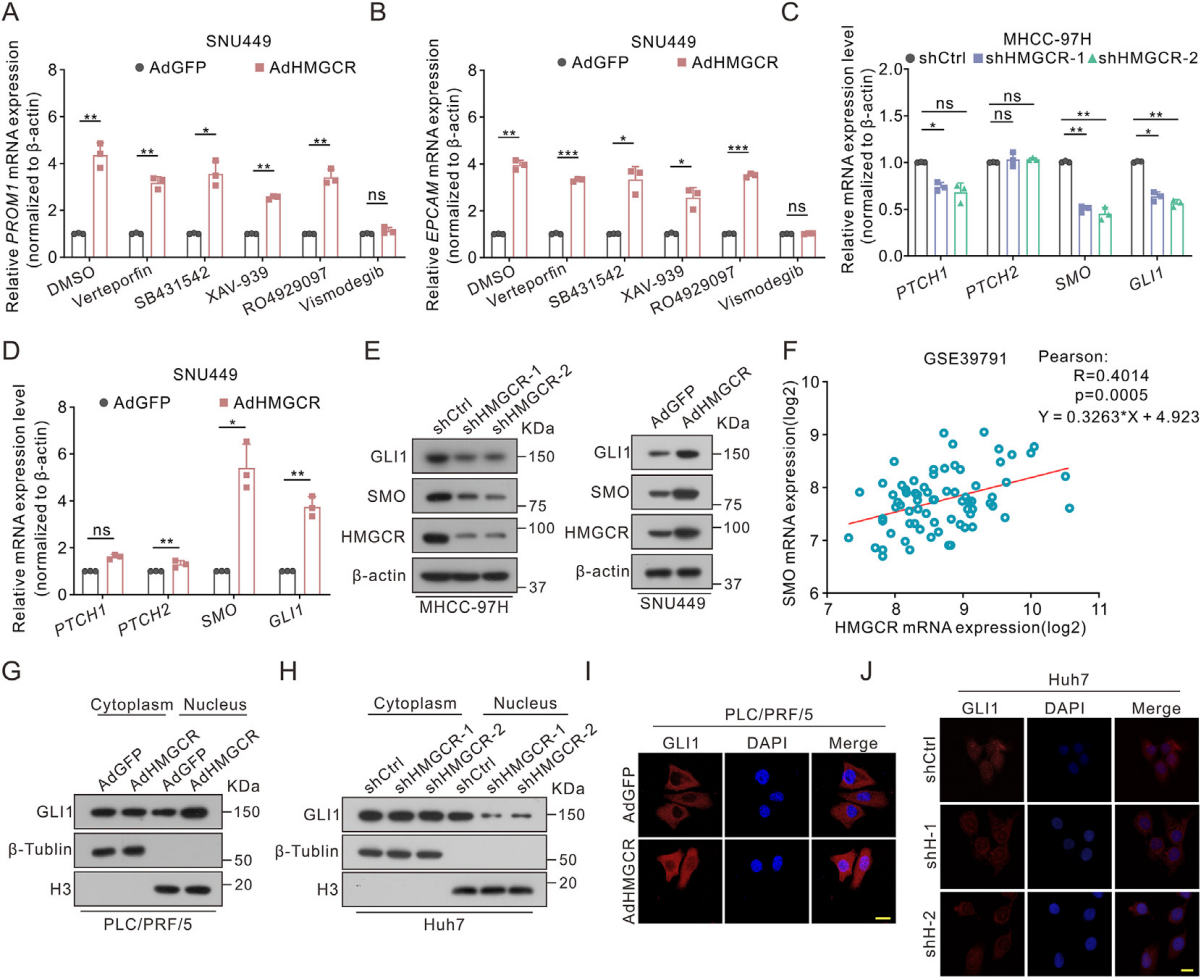
Pathway inhibitor screening revealed that HMGCR was a regulator of Hedgehog signaling. **(A, B)** Quantitative reverse transcription PCR for *PROM1* (A) and *EPCAM* (B) mRNA expression in AdGFP and AdHMGCR cells with multiple signaling inhibitor treatment for 36 h (5 μM for verteporfin and 10 μM for others). **(C, D)***PTCH1*, *PTCH2*, *SMO*, and *GLI1* mRNA levels were detected by quantitative reverse transcription PCR in HMGCR knock-down (C) or overexpression (D) hepatoma cells. **(E)** SMO and GLI1 protein levels in HMGCR knock-down MHCC-97H cells and overexpression SNU449 cells. **(F)** Correlation analysis between HMGCR and SMO in the GSE39791 dataset. **(G, H)** Immunoblot analysis of the GLI1 levels in nuclear and cytoplasmic fractions in hepatoma cells. β-Tubulin and H3 were used as cytoplasmic and nuclear fraction controls respectively. **(I, J)** Subcellular localization of GLI1 in hepatoma cells by immunofluorescence staining with TRITC. The nucleus was stained with DAPI. Scale bar: 20 μm. Data are shown as mean ± standard deviation. ns, not significant; ^∗^*P* < 0.05, ^∗∗^*P* < 0.01, ^∗∗∗^*P* < 0.001. Differences were tested using two-sample *t*-test for (A, B, D) and one-way ANOVA for (C).

**Figure 7:**
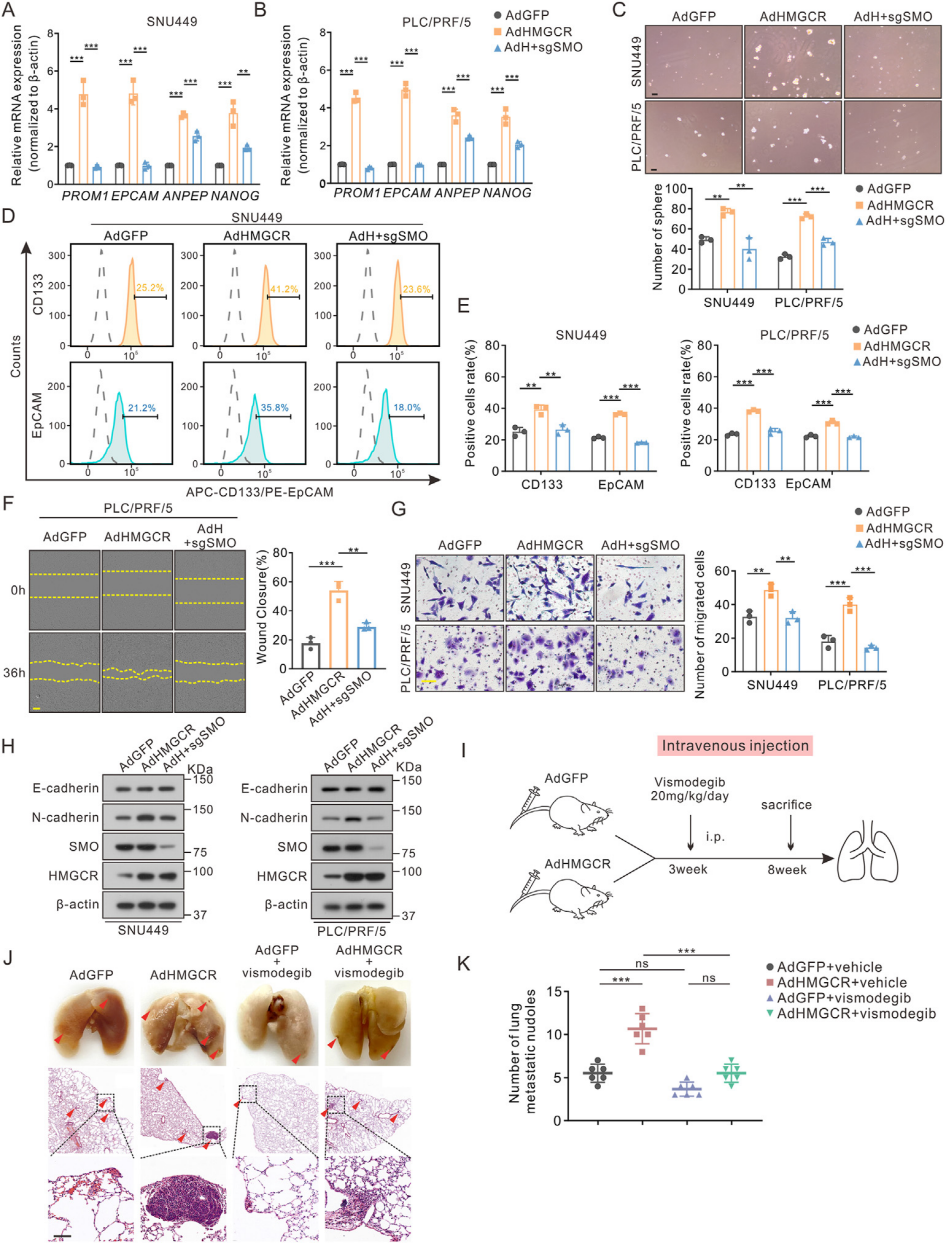
Suppression of Hedgehog signaling reversed the metastasis-promoting effects by HMGCR. All rescue assays here were divided into three groups (control, AdHMGCR, and AdHMGCR with sgSMO). **(A, B)***PROM1*, *EPCAM*, *ANPEP*, and *NANOG* in SNU449 and PLC/PRF/5 were tested by quantitative reverse transcription PCR. **(C)** Sphere formation assays in SNU449 and PLC/PRF/5 cells. Scale bar: 100 μm. **(D, E)** The population of CD133^+^ and EpCAM^+^ cells in hepatoma cells by flow cytometry. The statistical analysis was shown as percentages in (E). **(F, G)** Representative and quantified results of the wound-healing (F) and transwell assays (G) in hepatoma cells. Scale bar: 100 μm in transwell and 200 μm in wound-healing assays. **(H)** N-cadherin and E-cadherin expression in SNU449 and PLC/PRF/5 cells by western blotting. **(I–K)** The groups, treatment of tail intravenous injection model (I), and hematoxylin-eosin staining of lung tissues (J). Scale bar: 100 μm. The number of lung metastatic nodules is shown in (K) (*n* = 6). Data are shown as mean ± standard deviation. ns, not significant; ^∗∗^*P* < 0.01, ^∗∗∗^*P* < 0.001. Differences were tested using one-way ANOVA for (A–G, K).

### Statistical analysis

All the statistical analyses and graphical representations mentioned above were completed using GraphPad 8.0. Differences were considered significant at *P* < 0.05 (^∗^*P* < 0.05, ^∗∗^*P* < 0.01, ^∗∗∗^*P* < 0.001). Quantitative data were presented as mean ± standard deviation in figures. Unless particularly mentioned, all quantitative experiments were carried out with three independent replicates. A Student's *t*-test was used to compare the differences between two groups. As for multiple comparisons, statistical significance was determined using a one-way ANOVA analysis of variance. The HMGCR expression levels in HCC tissues and the adjacent non-tumor tissues were compared using paired *t*-test analysis. The assessment of overall survival was conducted utilizing the Kaplan–Meier method in conjunction with the log-rank test. Pearson's correlation coefficient was used to test the linear correlation. The tumor formation incidence from the *in vivo* limiting dilution assays was calculated using the extreme limiting dilution analysis website (https://bioinf.wehi.edu.au/software/elda/).

## Results

### HMGCR is up-regulated in HCC tissues and correlated with poor overall survival

In order to examine the association between the expression of HMGCR and the clinicopathological characteristics, as well as the prognostic indicators of HCC, TCGA LIHC dataset analysis found that HMGCR is up-regulated in HCC patients compared with healthy individuals (Fig. 1A). Furthermore, we observed a correlation between elevated HMGCR expression and an unfavorable prognosis in patients with HCC (Fig. 1B). In 42 pairs of liver tissues from clinical HCC patients, compared with adjacent nontumor tissues, the expression of HMGCR was also significantly higher in HCC tissues (Fig. 1C). Similarly, immunohistochemical analysis of 15 pairs of clinical HCC and adjacent nontumor tissue slices confirmed these findings (Fig. 1D; Fig. S1A). In summary, HMGCR is up-regulated in HCC, and high HMGCR expression is tightly associated with the aggravation of HCC.

### Hepatocellular carcinoma stem cells have higher HMGCR expression

The GEO database (GSE23034) was then examined, revealing that induced pluripotent hepatocytes exhibited higher levels of HMGCR expression compared with normally developed hepatocytes (Fig. 2A). In dataset GSE5975, patients with positive stemness marker EpCAM displayed a significant up-regulation of HMGCR (Fig. 2B). These findings implied a potential association between HMGCR expression and HCC stemness. Consequently, the protein and mRNA levels of HMGCR in SNU449, PLC/PRF/5, MHCC-97H, and Huh7 low-differentiated cells induced by sphere formation culture were elevated (Fig. 2C, D). Therefore, we rationally speculated that HMGCR is involved in the stemness regulation of HCC.

### HMGCR is necessary for maintaining liver CSC proliferation and self-renewal

Next, we examined the intrinsic expression of HMGCR in various HCC cell lines (Fig. S2A). Specifically, we selected Huh7 and MHCC-97H cells, which exhibited high levels of HMGCR expression, to establish knock-down cell models using short hairpin RNA targeting HMGCR (shHMGCR). Conversely, SNU449 and PLC/PRF/5 cells, characterized by low HMGCR expression, were subjected to HMGCR overexpression via AdHMGCR adenovirus. The confirmation of the successful establishment of HMGCR knock-down and overexpression cells was accomplished through western blotting (Fig. S2B, C). Following 10 days of sphere formation culture, the diameter of spheres in the shHMGCR group was significantly smaller than that in the shCtrl group (Fig. 3A; Fig. S2D); on the contrary, AdHMGCR led to larger spheres (Fig. 3B). Meanwhile, the mRNA expression of *EPCAM*, *PROM1*, *ANPEP*, and *NANOG* which are HCC stemness-related markers was regulated by HMGCR (Fig. 3C, D; Fig. S2E, F). Further, the HMGCR-induced expression of EpCAM and CD133 on the surface of hepatoma cells was also identified by flow cytometry (Fig. 3E–G; Fig. S2G). Additionally, *in vivo* limiting dilution analysis demonstrated that knocking down HMGCR significantly inhibited subcutaneous tumorigenic capacity and frequency (Fig. 3H, I; Fig. S2H). Taken together, these results suggest that HMGCR promotes liver CSC proliferation and self-renewal mainly by up-regulating EpCAM and CD133.

### HMGCR promotes cancer metastasis in HCC

Tumor stem cells have been observed to possess a heightened propensity for metastasis compared with non-stem tumor cells.^9^^,^^21^ Considering the impact of HMGCR on the stemness of HCC, we proceeded to investigate the potential influence of HMGCR on the metastatic capacity of hepatoma cells. Transwell and wound healing assays demonstrated a significant reduction in the migratory potential in the shHMGCR groups, whereas a promotion in HMGCR-overexpression groups compared with control groups (Fig. 4A–D). Notably, there exists a positive correlation between the expression of biomarkers of epithelial–mesenchymal transition and cancer stemness, which contributes to the maintenance of the renewal ability of CSCs.^9^^,^^21^^,^^22^ In HMGCR-overexpression hepatoma cells, expression of N-cadherin, a marker of epithelial–mesenchymal transition, was increased, while it was decreased upon HMGCR knockdown. However, there was no significant change in the expression of E-cadherin (Fig. 4E, F).

### Blocking HMGCR by simvastatin impairs self-renewal and migration of HCC

Given the important role of HMGCR in the maintenance of liver CSCs, we aimed to clarify whether pharmacological intervention by targeting HMGCR could effectively inhibit the renewal and metastasis of liver CSCs. As expected, the sphere formation assay confirmed that treatment with simvastatin, an HMGCR inhibitor, significantly hindered the formation of spheres (Fig. 5A) and blocked the expression of stemness markers including *EPCAM*, *ANPEP*, *PROM1*, and *NANOG* at the transcriptional level in a dose-dependent manner (Fig. 5B; Fig. S3A). Results in flow cytometry were consistent with previous assays (Fig. 5C; Fig. S3B).

Simvastatin also impeded the metastatic potential of hepatoma cells by transwell and wound healing assay *in vitro* (Fig. 5D, E; Fig. S3C), accompanied by a reduction in the expression of the N-cadherin (Fig. 5F). Finally, to assess the impact of simvastatin on the metastasis of HCC *in vivo*, a tail vein injection model was established. HMGCR overexpression facilitated pulmonary metastasis and simvastatin administration led to a suppression of this process (Fig. 5G–I). Collectively, these data implied that simvastatin has the potential to damage the maintenance of pluripotency and the promotion of metastasis in HCC.

### HMGCR activates Hedgehog signaling by translocating GLI1 to the nucleus

Several signaling pathways have been demonstrated to play a role in regulating the stemness characteristics of tumor cells, including Wnt/β-catenin, TGFβ, Notch, Hippo, and Hedgehog signaling.^8^ To probe the molecular basis underlying stemness maintenance of HCC, the mRNA level of *EPCAM* and *PROM1* in the context of HMGCR overexpression were tested under the treatment of different signaling pathway inhibitors. The inhibitors employed included verteporfin for the Hippo pathway, SB431542 for TGFβ signaling, XAV-939 for the Wnt signaling, and vismodegib, an SMO inhibitor of Hedgehog. Only vismodegib could reverse the stimulatory effects of HMGCR on *EPCAM* and *PROM1* expression, highly indicating that HMGCR may stimulate the stemness and metastasis of HCC cells through the Hedgehog signaling (Fig. 6A, B; Fig. S4A, B).

To further verify the regulation of the Hedgehog pathway by HMGCR, the expression of key molecules of this pathway, containing PTCH1, PTCH2, SMO, and GLI1 was assessed. HMGCR enhanced the expression of SMO and GLI1 on protein and transcriptional levels, while HMGCR knockdown suppressed their expression (Fig. 6C–E; Fig. S4C, D). Additionally, a positive correlation between HMGCR and SMO expression in HCC was confirmed in the GSE39791 dataset (Fig. 6F). As a transcriptional activator of the Hedgehog signaling target genes, GLI1 notably translocates into the nucleus and initiates the transcription of target genes related to cancer progression.^23^ Expectedly, HMGCR facilitated the nuclear translocation of GLI1, whereas HMGCR knockdown played an opposite role (Fig. 6G, H; Fig. S4E, F). Immunofluorescence staining also confirmed this conclusion (Fig. 6I, J). Altogether, our results suggest that HMGCR activates the Hedgehog signaling by up-regulating SMO and transporting GLI1 into the nucleus.

### HMGCR regulates self-renewal and migration of HCC via Hedgehog signaling

Next, we verified whether HMGCR influences the stemness and metastasis of HCC by modulating the Hedgehog signaling pathway. Here, results of stemness-related marker detection, sphere formation assays, and flow cytometry indicated that the down-regulation of SMO reversed the impact of HMGCR on the self-renewal ability of hepatoma cells (Fig. 7A–E; Fig. S5A). Furthermore, transwell and wound healing assays indicated that the promoting effect of HMGCR on cell metastasis could be counteracted by SMO deficiency (Fig. 7F, G; Fig. S5B). Knockdown of SMO also led to a decrease in N-cadherin levels which came from HMGCR overexpression (Fig. 7H). The tail vein injection model with the treatment of vismodegib targeting SMO provided further confirmation that HMGCR promoted the metastasis of HCC through the Hedgehog signaling *in vivo* (Fig. 7I–K). In conclusion, the influence of HMGCR on the stemness and metastasis of HCC is achieved through the regulation of the Hedgehog pathway.

## Discussion

In our present study, we focused on HMGCR, a molecule with elevated expression in HCC, which was associated with poor prognosis of HCC and was involved in maintaining the stemness of liver CSCs through database analysis. Functional assays clarified that HMGCR was essential for liver CSCs self-renew ability. Meanwhile, HMGCR facilitated the metastasis of HCC *in vivo* and *in vitro*. Through pathway inhibitor screening, we found that HMGCR promoted the stemness and metastasis of HCC by activating the Hedgehog signaling. Mechanistically, HMGCR up-regulated SMO level and translocated GLI1 into the nucleus from the cytoplasm. All of these indicated that HMGCR may be a gene vulnerability of liver CSCs.

As the mechanistic link between up-regulation HMGCR and stemness of metastatic HCC, Hedgehog signaling has shown promising restraint in the progression of malignant tumors. A previous study found that after passing through transwell selection, CD133^−^/EpCAM^−^ Huh7 cells gained high expression of matrix metalloproteinases and GLI 1 compared with the native Huh7 cells. SMO inhibitors partially suppressed the expression of matrix metalloproteinases and GLI1 and attenuated their invasive behavior, indicating that the metastatic behaviors are under the control of the Hedgehog signaling pathway.^24^ Another research indicated that activation of Hedgehog signaling by CHSY1 promoted the stemness and metastasis phenotype of HCC.^25^ There is a similar finding in gastric cancer demonstrating that HMGCR facilitates migration through Hedgehog signaling.^26^ Consistently, our present study also confirmed that Hedgehog signaling was activated by HMGCR to regulate stemness and metastasis of HCC.

Our speculation about the specific molecular mechanism under the regulation of the Hedgehog signaling pathway by HMGCR involves two ways. The first is associated with cholesterol. The Hedgehog pathway can be regulated by cholesterol hydroxyl derivatives, including but not limited to 24(S),25-epoxycholesterol, 7-keto-25-hydroxycholesterol, 7-keto-27-hydroxycholesterol, 7β, 25-dihydroxy cholesterol, and 7β, 27-dihydroxy cholesterol. They activate Hedgehog signaling by interacting with receptors PTCH and SMO in the forms of binding, post-modification, or others.^27^^,^^28^ Moreover, cholesterol synthesis has been strongly certified to regulate the stemness of tumor cells in breast cancer, bladder cancer, and colon cancer.^13^, ^14^, ^15^ Therefore, we can reasonably infer that the most possible regulatory mechanism is that high expression of HMGCR promotes the synthesis of cholesterol hydroxyl derivatives and subsequently facilitates the activation of SMO. In addition, the prenylation modification mediated by the downstream metabolite GGPP is likely to take responsibility for the regulation of the Hedgehog pathway.^29^ In brief, cholesterol synthesis may be the major contributor to Hedgehog signaling regulated by HMGCR, additional investigation is necessary to confirm our conjecture.

Statins are the most widely used cholesterol-lowering drugs by targeting HMGCR in clinical practice. Besides, the pleiotropic effects of statins have been widely documented, with both preclinical^30^, ^31^, ^32^, ^33^, ^34^, ^35^, ^36^, ^37^, ^38^ and clinical^39^, ^40^, ^41^, ^42^, ^43^ studies demonstrating their anti-tumor impact in multiple ways. In cancers, administration of statin-induced oxidative stress accumulation and apoptosis through the GGPP synthase 1-RAB7A-autophagy axis in small-cell lung cancer.^31^ As for melanomas, pitavastatin prevented the production of GGPP and the prenylation of the Rab family, thus inhibiting tumor cell proliferation by the integrin/pFAK axis.^32^ Collectively, interfering with the prenylation of proteins by suppressing the production of GGPP is the main mechanism underlying statins' anti-tumor effects. Notably, the inhibitory effect of statins on tumor stemness and metastasis has also been reported in breast, ovarian, and pancreatic cancers.^44^, ^45^, ^46^ In HCC, clinical investigations have certified that the administration of statins can effectively lower the risk of HCC and alleviate the unfavorable prognosis.^47^^,^^48^ The beneficial effects of statins were more prominent in HCC with microscopic vascular invasion or early HCC recurrence after resection.^49^ Our findings also confirmed that simvastatin can hinder metastasis by inhibiting the stemness of hepatoma cells.

Nevertheless, several studies considered that the administration of statins may lead to an up-regulation of HMGCR expression and form a negative feedback regulation. This means the simultaneous use of a combination strategy to enhance the anti-cancer efficacy of statins is necessary.^50^^,^^51^ In addition, Dorsch et al^52^ discovered that statin could prevent tumor cells from mesenchymal-to-epithelial transition and metastasis formation, but it promotes metastatic seeding. This implies that prolonged and high-dose administration of statins is crucial for curative effect which is consistent with the conclusion of another study.^43^^,^^53^ Further investigation is required to ascertain the beginning stage, dosage, and combination treatment strategy of statins' clinical application.

**Figure 8:**
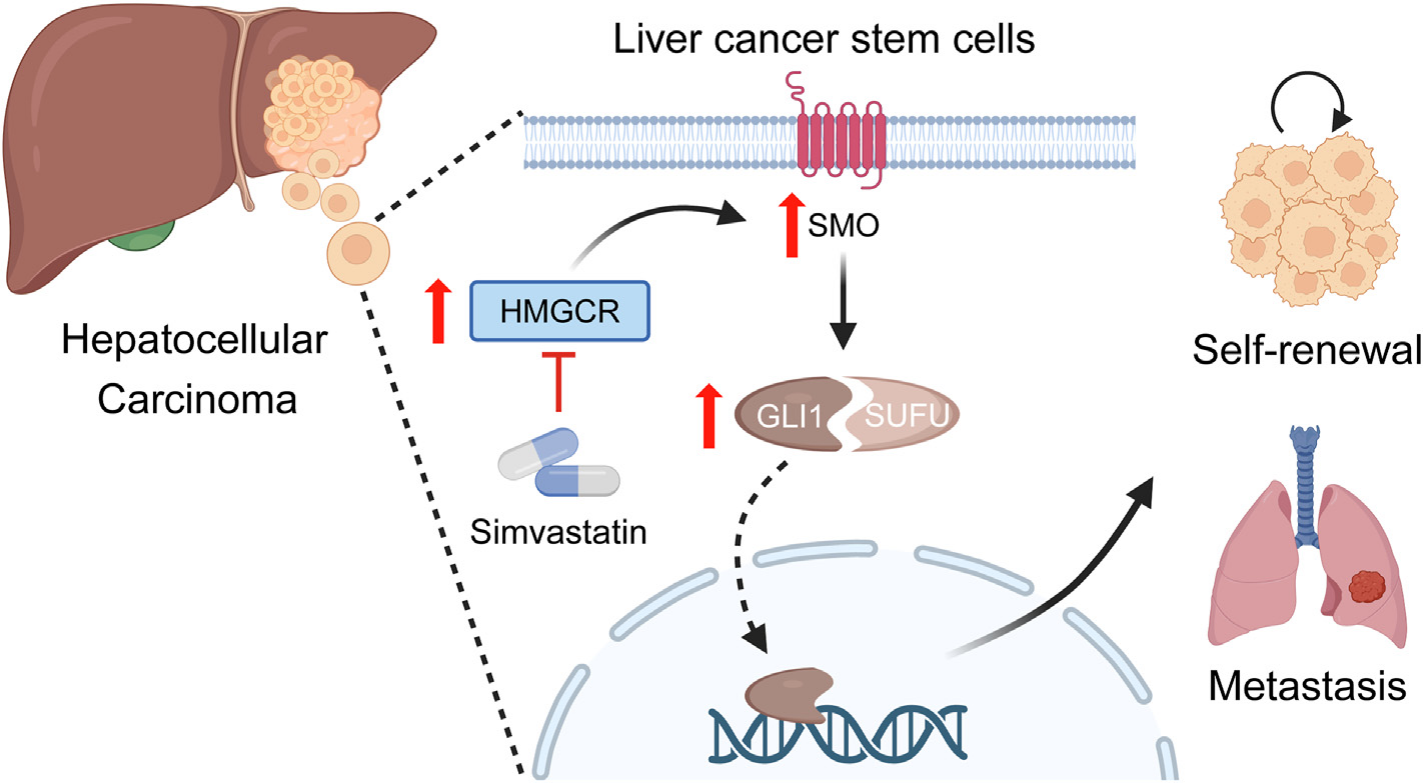
Working summary of the study. Schematic depiction of the underlying mechanism that HMGCR activates the Hedgehog signaling pathway to promote self-renewal and metastasis of hepatocellular carcinoma.

In summary, our study uncovered that HMGCR contributes to the stemness and metastasis of HCC by stimulating SMO expression and transporting GLI1 into nuclear in the Hedgehog signaling pathway. Also, the potential therapeutic role of simvastatin in suppressing the recurrence and metastasis of HCC was stressed (Fig. 8). It provided an efficient way to prevent the distant metastasis of HCC from the origin by targeting liver CSCs. The long-term safety application of statins makes it more feasible for patients who suffer from HCC. In the future, optimizing the therapeutic effectiveness of statins, carefully developing a synergistic strategy, and establishing an appropriate medication regimen are essential.

## Author contributions

Zhirong Zhang: conceptualization, methodology, validation, formal analysis, data curation, writing - original draft, and writing - review & editing. Jiayao Yang: validation, methodology, and investigation. Rui Liu: investigation and methodology. Jin Ma: investigation and formal analysis. Ni Tang, Xiaojun Wang, and Kai Wang: resources, project administration, supervision, funding acquisition, and writing - review & editing.

## Conflict of interests

The authors declared no conflict of interests.

## Funding

This work was supported by the National Key Research and Development Program of China (No. 2023YFC2306803), the China National Natural Science Foundation (No. 82273238, 82272975, 82072286), the Innovative and Entrepreneurial Team of Chongqing Talents Plan, Chongqing Medical Scientific Research Project (Joint Project of Chongqing Health Commission and Science and Technology Bureau; No. 2023DBXM007), Senior Medical Talents Program of Chongqing for Young and Middle-aged, the Kuanren Talents and DengFeng program of the Second Affiliated Hospital of Chongqing Medical University (China), the Future Medical Youth Innovation Team of Chongqing Medical University (China) (No. W0036, W0101), and the Postdoctoral Fellowship Program of China Postdoctoral Science Foundation (No. GZC20233349).

## Data availability

The data that support the findings of this study are available from the corresponding author upon reasonable request.
