# Supplementary material for: Inhibiting HMGCR represses stemness and metastasis of hepatocellular carcinoma via Hedgehog signaling
Source: Genes Dis. 2024 Apr 3;11(5):101285. doi: 10.1016/j.gendis.2024.101285 (PMC11252768; doi:10.1016/j.gendis.2024.101285)
Supplement: Multimedia component 1 [file mmc1.docx]

**Supplementary Material**

**Inhibiting HMGCR Represses Stemness and Metastasis of Hepatocellular Carcinoma via Hedgehog Signaling.**

Zhirong Zhang^a,1^, Jiayao Yang^a,1^, Rui Liu^a,1^, Jing Ma^a^, Kai Wang^a,^*, Xiaojun Wang^b,^*, Ni Tang^a,^*

**Supplementary Figures.**

**Supplementary Table S1.** **Sequence for primers.**

**
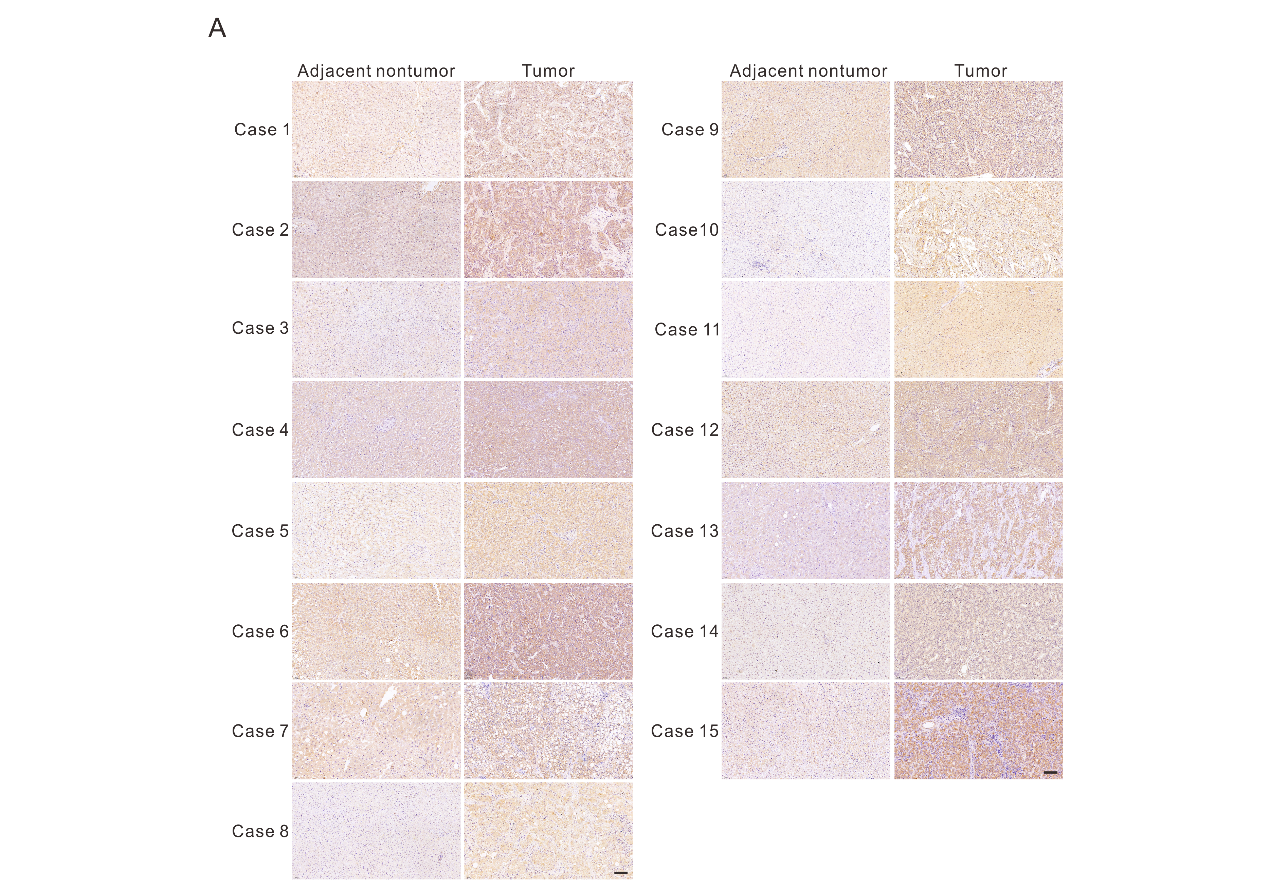
**

**Figure S1** HMGCR is highly expressed in HCC. **(A)** Complete images of IHC staining of HMGCR in a cohort of 15 HCC patients. Scale bar: 100μm.

**
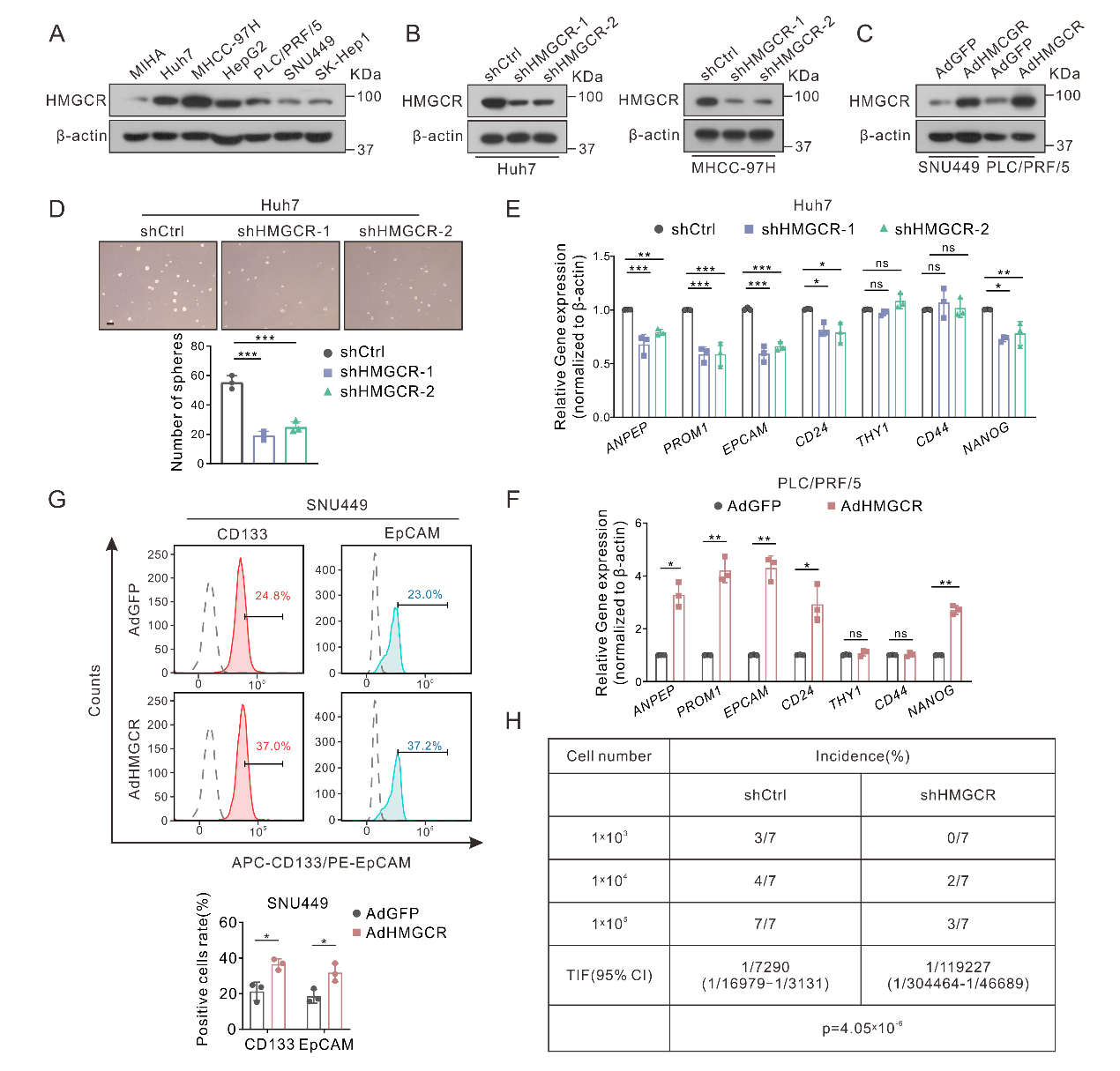
**

**Figure S2** HMGCR promoted HCC stemness features. **(A)** Endogenous HMGCR protein expression in HCC cell lines; **(B, C)** shHMGCR knock-down (B) and AdHMGCR overexpression (C) efficacy were evaluated by western blotting; **(D)** Representative images of Huh7 spheres after 10 days sphere formation culture and the number of hepato-spheres larger than 70μm in diameter. Scale bar: 100μm; **(E, F)** Liver CSC markers determined by qRT-PCR in HMGCR knock-down and overexpression cells; **(G)** The populations of CD133^+^ and EpCAM^+^ in HMGCR-overexpressing SNU449 cells by flow cytometry. The statistical analysis was shown as percentages; **(H)** Calculation table of tumor incidence frequency of *in vivo* limiting dilution xenograft formation; Data shown as mean ± SD. ns not significant, **P*<0.05, ***P*<0.01, ****P*<0.001. Differences were tested using one-way ANOVA for (D, E) and two sample *t*-test for (G, F).

**
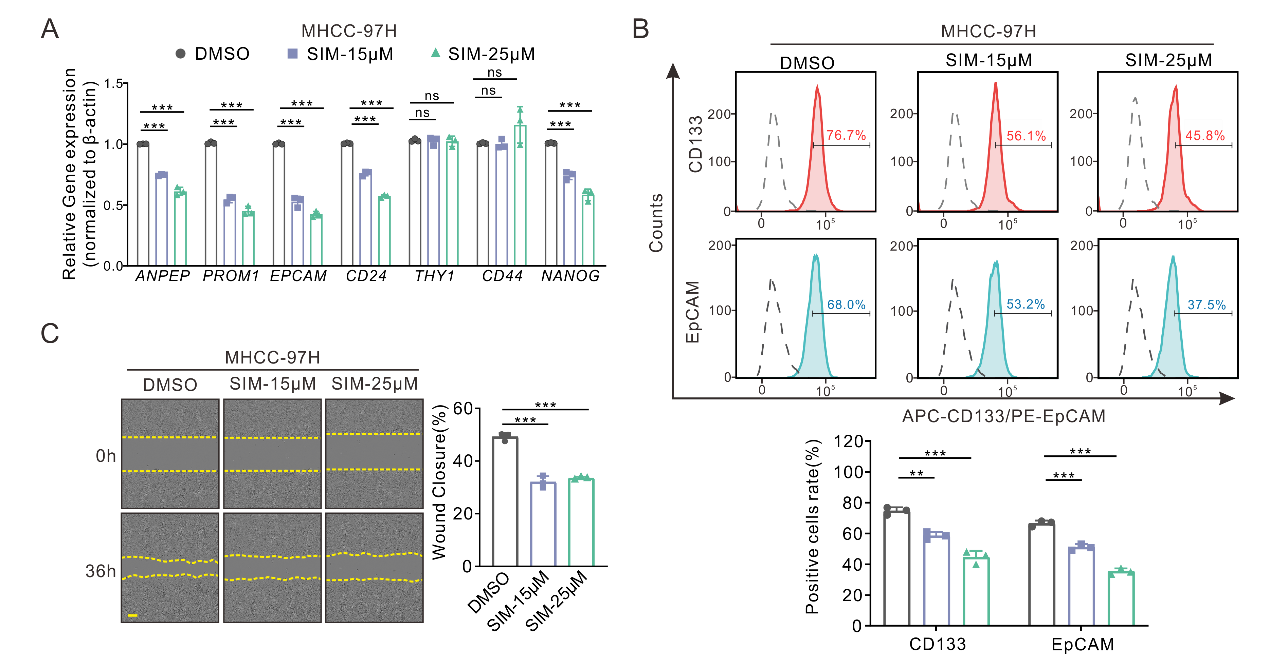
**

**Figure S3** Pharmacological inhibition of HMGCR impaired stemness and metastasis of hepatoma cells. **(A)** Stemness-related markers in simvastatin-treated MHCC-97H cells were quantified by qRT-PCR; **(B)** Flow cytometry for the populations of CD133^+^ and EpCAM^+^ MHCC-97H cells treated with simvastatin; **(C)** Representative images and quantified results of the wound-healing assay in simvastatin treated MHCC-97H cells. Scale bar: 200μm; Data shown as mean ± SD. ns not significant, ***P*<0.01, ****P*<0.001. Differences were tested using one-way ANOVA for (A-C). SIM: simvastatin.

**
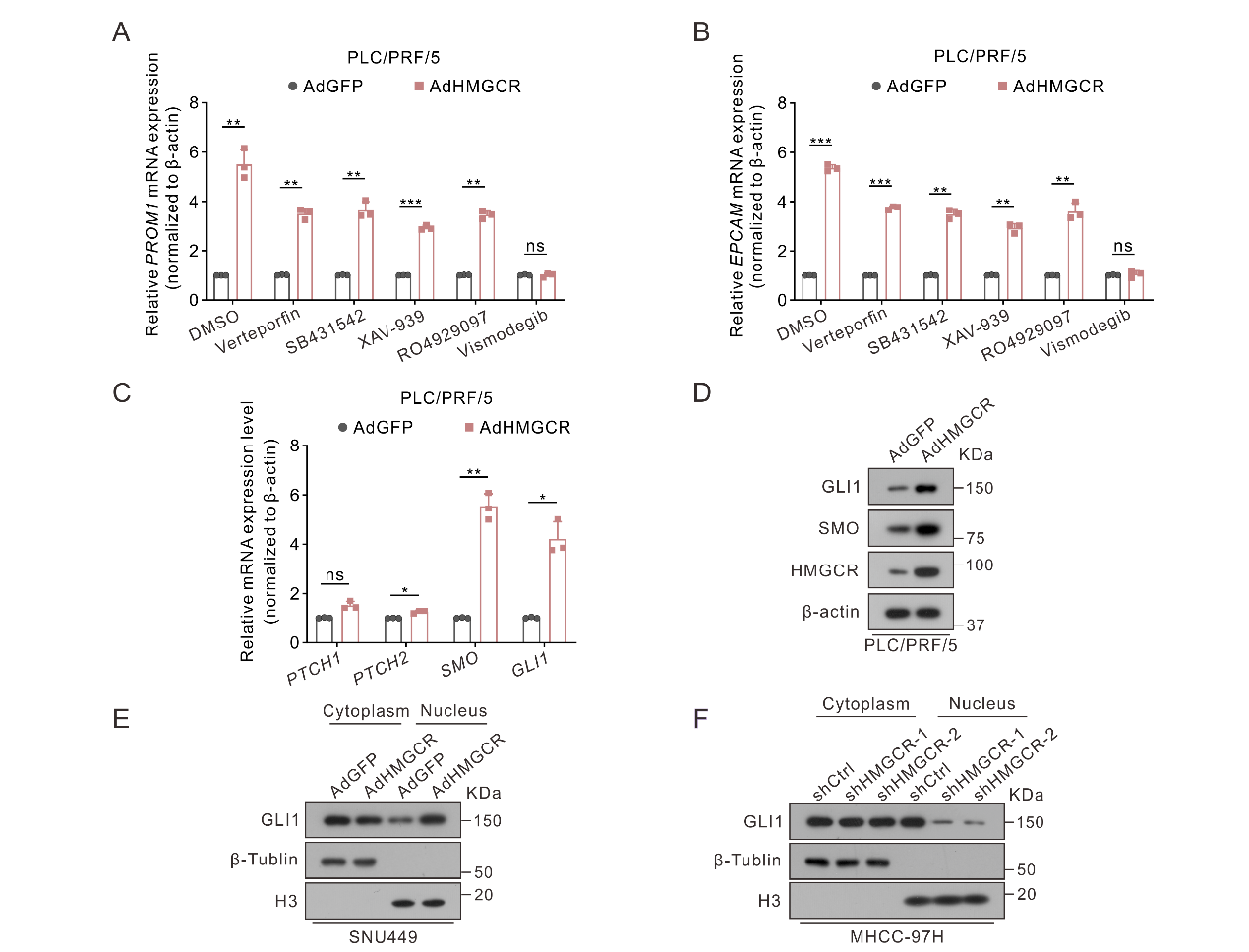
**

**Figure S4** Pathway inhibitors screening revealed that HMGCR was a regulator of Hedgehog signaling. **(A, B)** *PROM1* (A) and *EPCAM* (B) mRNA levels in AdGFP and AdHMGCR cells with multiple signaling inhibitors treatment; **(C)** *PTCH1, PTCH2, SMO, GLI1* mRNA levels in HMGCR-overexpression PLC/PRF/5 cells; **(D)** Western blotting for GLI1 and SMO in HMGCR-upregulated PLC/PRF/5 cells; **(E, F)** Immunoblot analysis of the GLI1 levels in nuclear and cytoplasmic fractions in SNU449 and MHCC-97H; Data shown as mean ± SD. ns not significant, **P*<0.05, ***P*<0.01, ***P<0.001. Differences were tested using two sample *t*-test for (A-C).

**
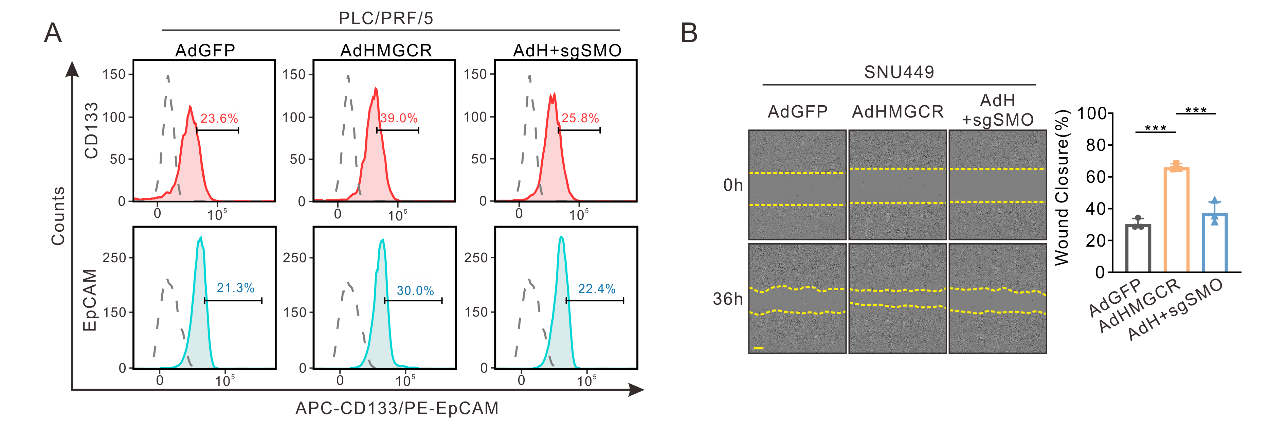
**

**Figure S5** Suppression of Hedgehog signaling reversed the metastasis-promoting effects by HMGCR. All rescue assays here were divided into three groups of control, AdHMGCR, and AdHMGCR with sgSMO. **(A)** Flow cytometry for the population of CD133^+^ and EpCAM^+^ PLC/PRF/5 cells; **(B)** Representative images and statistical analysis of wound-healing assay for SNU449 cells. Scale bar: 200μm; Data shown as mean ± SD, ***P<0.001. Differences were tested using one-way ANOVA for (B).

**Table S1** Sequence of primers.

| Primer | Strand | Sequence (5’-3’) |
| --- | --- | --- |
| Sequence for sub-clone | | |
| HMGCR | sense | TGC GGATCC ACC ATG GGC TTGTCAAGACTTTTTCGAAT |
|  | antisense | ATCTGAGTGGGTCTGGAGGT |
| Sequence for sgRNA or shRNA | | |
| shHMGCR#1 | sense | TGGAAAATATTGCTCGTGGTTCAAGAGACCACGAGCAATATTTTCCTTTTTTC |
|  | antisense | TCGAGAAAAAAGGAAAATATTGCTCGTGGTCTCTTGAACCACGAGCAATATTTTCCA |
| shHMGCR#2 | sense | TGGTCAAGATGATTATGTCTTCAAGAGAGACATAATCATCTTGACCTTTTTTC |
|  | antisense | TCGAGAAAAAAGGTCAAGATGATTATGTCTCTCTTGAAGACATAATCATCTTGACCA |
| sgSMO | sense | CACCGGTATAGTGACTGGTAGGAA |
|  | antisense | AAACTTCCTACCAGTCACTATACC |
| Sequence for quantitative real-time reverse transcription PCR | | |
| *HMGCR* | sense | CAAACATTGTCACCGCCATC |
|  | antisense | CCACCCACCGTTCCTATCTC |
| *ANPEP* | sense | CCTGTCCGAGGACGTATTCA |
|  | antisense | TCTGAGGGGCGGGTAACAT |
| *PROM1* | sense | CTGATGCCTCTGGTGGGG |
|  | antisense | ACGCCTTGTCCTTGGTAGTGT |
| *EPCAM* | sense | GTTCGGGCTTCTGCTTGC |
|  | antisense | GCCATTCATTTCTGCCTTCAT |
| *CD24* | sense | ATGGGCAGAGCAATGGTGG |
|  | antisense | GCCTTGGTGGTGGCATTAGTT |
| *THY1* | sense | CTAGTGGACCAGAGCCTTCGT |
|  | antisense | GAGGACCTTCATGTTGTATTTGC |
| *CD44* | sense | GGACTCTGCCTCGTGCCG |
|  | antisense | CGTGCCCTTCTATGAACCCA |
| *NANOG* | sense | GCCGAAGAATAGCAATGGTG |
|  | antisense | CAGGAGAATTTGGCTGGAAC |
| *PTCH1* | sense | CTGGCAGGAGGAGTTGATTG |
|  | antisense | AGTGCTCGTACATTTGCTTGG |
| *PTCH2* | sense | TTTCGCCCGCTATCAGTTT |
|  | antisense | AGGCACCACATCCGTCAGG |
| *SMO* | sense | TCTCGGGCAAGACCTCCTA |
|  | antisense | CCCACAAAACAAATCCCACTC |
| *GLI1* | sense | CCTTCCTACCAGAGTCCCAAGT |
|  | antisense | GCCCTATGTGAAGCCCTATTT |
